# H5N1 Virus Evolution in Europe—An Updated Overview

**DOI:** 10.3390/v1031351

**Published:** 2009-12-23

**Authors:** Giovanni Cattoli, Alice Fusaro, Isabella Monne, Ilaria Capua

**Affiliations:** OIE, FAO and National Reference Laboratory for Avian Influenza and Newcastle Disease, Istituto Zooprofilattico Sperimentale delle Venezie, Viale dell’Università 10, 35020 Legnaro, PD, Italy

**Keywords:** Europe, H5N1, avian influenza

## Abstract

Since its emergence in South East Asia in 2003, Highly Pathogenic Avian Influenza (HPAI) A/H5N1 has reportedly caused outbreaks in poultry and/or wild birds in 62 countries, of which 24 were in Europe. Interestingly, out of the many genetic clades circulating in Asia, the westward spread of HPAI A/H5N1 to Central Asia, the Middle East, Europe and Africa was dominated by one single clade, namely clade 2.2. In this paper, we review and update through phylogenetic and gene migrational analysis the information concerning the evolution and the molecular epidemiology of HPAI A/H5N1 on the European continent.

## 1. Introduction

The highly pathogenic avian influenza (HPAI) virus belonging to the A/H5N1 subtype was detected for the first time in geese in Guangdong province of China in 1996 [1]. Since then, this virus spread significantly in terms of hosts and geography. HPAI A/H5N1 has been described in a wide variety of species which were not before considered as susceptible species for the highly pathogenic form of the disease, including wild and domestic waterfowl and mammals, such as humans, felines, dogs, civets, mink and stone marten. In 1996–1997 the circulation of this virus appeared to be limited to poultry in South Eastern China and Hong Kong at the farm level or in live bird markets. However, in 1997 the first avian to human transmission of HPAI A/H5N1 virus was detected in Hong Kong causing 18 cases, of which six were fatal [2]. In spite of the strict control measures taken the virus persisted and evolved in the region causing several outbreaks in poultry, wild birds and humans [3–4] between 2001 and 2003. The appearance of several distinct genotypes through multiple reassortment events [5] was a natural consequence of extensive circulation of the virus. A dominant reassorted genotype, named genotype Z [6], emerged at an unprecedently wide geographical scale in late 2003. Recent studies indicate that this genotype probably originated in China in late 1999 or early 2000 [7]. These viruses continued to evolve, such that Z genotype viruses revealed genetic and antigenic differences and based on the current nomenclature [8], they are now distributed into several distinct genetic clades [7]. From December 2003 to February 2004, HPAI A/H5N1 outbreaks were almost simultaneously detected in Far East Asia, namely the Republic of Korea, Thailand, Japan, Cambodia, Lao PDR and Indonesia [4], mainly in poultry, but also in some wild birds, felines and humans.

The observations and the studies carried out on this virus since its emergence have raised immediate concerns about its devastating impact on the poultry economy and, above all, on the serious veterinary and public health implications. Nevertheless, for about eight years (1997–2004) this infection was perceived as a major problem for Eastern Asian countries only and most of the control efforts, surveillance programs and research investigations focussed on this area of the globe.

This scenario dramatically changed in April 2005, when HPAI A/H5N1 virus was demonstrated to be the cause of the massive mortality registered in wild birds that had congregated at Qinghai Lake in North-Western China [9]. A few months later (July 2005) the virus appeared in Russian Siberia and Kazakhstan [10], moving westward and reaching Central Asia, the Middle East, Europe and Africa within a few months [11].

Since its re-emergence in 2003, HPAI A/H5N1 has been reported as causing outbreaks in poultry and/or wild birds in 62 countries on three continents of which 24 are located in Europe [12]. Concurrently, the number of cases in humans increased both in the originally infected Asian countries, such as China and Indonesia and in the newly infected areas of Asia and Africa. To date, none of the 438 registered human cases have come from the European continent [13].

The aim of the present paper is to review and update the information concerning the evolution and the molecular epidemiology of HPAI A/H5N1 with particular reference to the European continent.

## 2. The emergence of divergent genetic clades

As for other type A influenza viruses, HPAI A/H5N1 has an impressive capacity of mutating and evolving over time, changing its genetic and antigenic characteristics. The prolonged and continuing circulation of HPAI A/H5N1 in different geographical areas and hosts has led to an increase in the number of isolates which have been genetically sequenced. As a consequence, several distinct genetic groups or clades have been identified. Initially, these clades were distinguished by names or codes given by the single research team that described them first, creating some confusion in the interpretation of the molecular epidemiology of this virus. In 2008, under the auspices of the WHO/OIE/FAO, a review of the clade definition aiming at a unified nomenclature was launched [8]. The system is based on the genetic characterization and sequence homology of the hemagglutinin (HA) gene and clades are identified according to well defined criteria. Clades are coded by Arabic numbering (e.g. clade 1; clade 2; *etc.*) [8]. However, since HPAI A/H5N1 continues to evolve, new virus clades are expected to emerge periodically. These new clades are defined, according to the same specific criteria, as second or third order clades and assigned a numerical address which links them to their original clade number (e.g. clade 2.2 or clade 2.3.1). In addition, distinct genetic clusters may emerge that do not meet the defined criteria for clade designation, at least at their initial stage of evolution. Even though these genetic clusters can provide important information related to the epidemiology of the virus and its evolution, they should not be identified with the same numerical address system described above until that sublineage has met each of the specific criteria for HA clade definition. This should avoid confusion and overlapping nomenclature [14].

To date, ten first order clades have been identified, numbered from 0 to 9. Clade descriptions have been provided in previous publications [8–14].

Interestingly, of the many clades circulating in Asia, the westward spread of HPAI A/H5N1 from East Asia to Central Asia, the Middle East, Europe and Africa between 2005–2008 was dominated by one single clade, namely clade 2.2, or the EMA (Europe-Middle East-Africa) clade as initially identified [11]. The prototypes of clade 2.2 were the viruses responsible for the massive mortality of wild birds registered in April/May 2005 at Qinghai lake [13], where more than 6,000 wild birds died, mainly bar-headed geese (*Anser indica*) [9]. The HPAI A/H5N1 viruses responsible for this outbreak in 2005 exhibited a certain degree of genetic heterogenicity and at least four distinct genotypes (A to D) were identified [9]. Over time, clade 2.2 has further diversified. A third order clade, denominated clade 2.2.1 [14], and three distinct genetic sublineages, namely 2.2 sublineage I, II and III [15] can now be distinguished.

## 3. The westward spread of clade 2.2

In July and August 2005, clade 2.2 viruses were detected in small, rural poultry flocks in Western Siberia (Novosibirsk region) and Kazakhstan and in wild birds in Mongolia [3,4,9,16]. These viruses were reported as being closely related to the viruses responsible for the Qinghai lake outbreak [9–17].

The infection then moved further west within Russia [18] and, to date, a total of 149 outbreaks in poultry plus other cases in wild birds have been notified in the Russian Federation [12]. During October 2005, the HPAI A/H5N1 virus was reported in a turkey farm in Western Turkey and in backyard poultry in the Danube delta, Romania, almost simultaneously [19–20]. Croatia was the first Central European country to register an HPAI A/H5N1 outbreak in wild birds [mute swans (*Cygnus olor*)], on the 21 October 2005 while in December 2005, the Ukraine reported cases in poultry. Prior to 2006 Western Europe had only experienced two HPAI A/H5N1 introductions which were through the illegal and legal importation of birds from Eastern Asia to Belgium and the United Kingdom, in 2004 and 2005 respectively [21–22]. These cases, caused by viruses not belonging to clade 2.2, were immediately detected and contained at the EU border with no serious consequences. From the beginning of 2006, infected wild birds, mainly swans, were detected in an increasing number of European countries, from East to West, North to South. During the same period of time (*i.e.,* the end of 2005/beginning of 2006) HPAI A/H5N1 clade 2.2 virus was also notified in wild birds and/or poultry in Middle Eastern countries and in Africa [12].

In Europe, the HPAI A/H5N1 infection was largely confined to the wild bird population and only 13/24 infected European countries reported the presence of the virus in poultry, in either backyards or commercial flocks. In many instances the infection of poultry was limited to one to 10 outbreaks, with the exception of Russia, Romania and the Ukraine (Table 1) [12].

## 4. Genetic diversity of HPAI A/H5N1 viruses in Europe

In Europe, the reporting of cases in wild birds and, more sporadically, in poultry continued throughout 2006 and 2007 (Table 1). Outbreaks apparently declined in 2008 and during 2009, with the last incursion of the virus detected in wild birds in Germany (Bavaria, March 2009) and Eastern Russia (Tyva Republic, June 2009).

The global spread of HPAI A/H5N1 and the related concerns about its devastating potential on economy and public health, prompted the development of initiatives aiming to share knowledge and data on this virus. These included the submission of virus gene sequences in public databases [23] under the auspices of many international organizations such as WHO/FAO/OIE and the publication of several reports on this virus worldwide, in some cases in open access scientific journals.

This situation is well reflected in Europe, where complete or partial virus genetic data [at least concerning the major antigenic and virulence determinant, *i.e.,* the hemagglutinin (HA)] are available in the public domain for 19 out of 24 countries that have experienced HPAI A/H5N1 cases (GenBank, http://www.ncbi.nlm.nih.gov/, accessed 22 July 2009; GISAID http://platform.gisaid.org/, accessed 4 August 2009).

At the time of writing, detailed molecular and phylogenetic information concerning HPAI A/H5N1 in Europe have been published in internationally available scientific journals concerning isolates detected in 2004–2007 in Belgium [21], Russia [16–17], Italy, Croatia, Slovenia [11], Denmark [24], Germany [25–29], France [30], the Czech Republic and Slovakia [31,32], Great Britain [33], Sweden [34], Spain [35]; Switzerland [36] and Hungary [37].

Based on the above cited reports, in many European countries multiple, distinct introductions of clade 2.2 viruses were detected during 2006 and 2007, similar to what has been described for Africa [11,15,38]. In fact, the introduction of at least 2 groups of phylogenetically distinguishable clade 2.2 viruses was evident in 2006 in Germany [26–27], France [30], Sweden [34], Italy [11], the Czech Republic [32] and Hungary [37]. An additional genetic distinct lineage, previously detected only in Russia and Italy in 2006, was subsequently introduced into Central Europe in 2007, as publications from Germany [27], the Czech Republic [32] and Hungary [37] reported.

## 5. Updating the molecular epidemiology of HPAI A/H5N1 in Europe

### 5.1. Phylogeny

At present a relevant amount of information concerning HPAI A/H5N1 in Europe is available in the public domain. However, data and information are not always provided in a harmonized manner. For example, the nomenclature may vary depending on the publication. Similarly, variations in the batch of sequences included in the phylogenetic study performed in distinct investigations and in the methods applied for their analysis are present. Furthermore, studies conducted to date included sequences from 2006 and 2007. At the time of writing, phylogenetic studies on 2008–2009 European viruses have not been published.

With the aim of providing an updated and comprehensive overview on the molecular epidemiology of HPAI A/H5N1 in Europe, we have downloaded from the publicly accessible database (GenBank, http://www.ncbi.nlm.nih.gov/) and aligned 190 sequences related to the complete HA gene of viruses isolated on the European continent from 2004 to 2009. Additionally, 63 representative Asian sequences and ten representative African sequences were aligned and phylogenetically compared. Maximum likelihood (ML) tree was estimated using the best-fit general time-reversible model (GTR) + I (proportion of invariant site) + γ_4_ (gamma distribution among site rate variation) model of bases substitution using PAUP* [39]. For the purpose of this study, sublineages within clade 2.2 that do not meet the established criteria for clade designation are identified according to a paper recently published, *i.e.,* clade 2.2 sublineage I, II and III [15].

At the time of writing, genetic sequences of HPAI A/H5N1 viruses representative of all but five (Albania, Bosnia Herzegovina, Bulgaria, Greece and Serbia Montenegro) European infected countries were available. The number of HA gene representative sequences per country varied from one sequence (*i.e.,* Belgium, Croatia, Spain) up to 64 (Germany) and 79 (Russian Federation).

**Figure 1.**
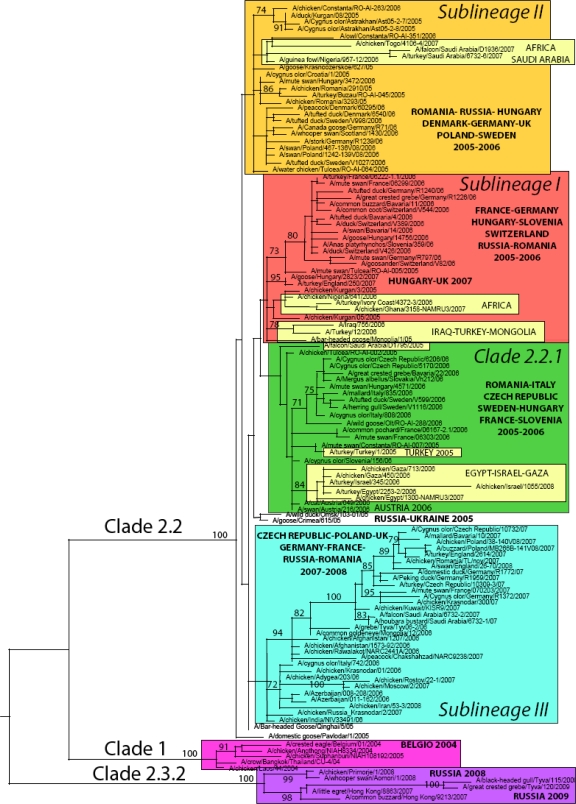
Maximum likelihood phylogenetic tree of the HA gene of representative H5N1 avian influenza viruses sampled in Europe, Asia, Africa and the Middle East. Coloured rectangles represent three clades (1, 2.2.1 and 2.3.2) and three distinct sublineages of 2.2 clades (sublineages I, II and III). Bootstrap values (>70) are shown for key nodes. All horizontal branch lengths are drawn to a nucleotide substitution per site scale.

According to the latest HPAI A/H5N1 nomenclature [14], five major virus genetic clusters can be identified in Europe between the period 2004–2009 (Figure 1). As mentioned earlier, the first introduction in Europe of HPAI A/H5N1 clade 1, was linked to the illegal importation of a Thai Eagle (*Spizaetus nipalensis)* in Belgium in 2004 [21] and was restricted to this event only. In 2005, an incursion of a clade 2.3 virus was identified in the UK [22]. In this case too, the event was isolated and occurred at the UK quarantine station in exotic birds imported from Suriname and Taiwan .

Viruses detected in wild birds and poultry in 2005 in Russia and Romania belonged to three distinct clades or sublineages, namely clade 2.2.1, clade 2.2 sublineage I and II. (Table 2). Only viruses belonging to clade 2.2 sublineage II were reported in 2005 from wild birds in Croatia (Figure 1, Table 2).

As illustrated in Figure 1 and summarized in Table 2, during 2006 the co-circulation in Europe of the three genetic groups that appeared in 2005 is evident for some countries, such as Germany, Hungary and Romania, while in other countries only one or two of these sublineages were present; confirming previous findings.

Interestingly, a fourth distinct genetic group (clade 2.2 sublineage III, Table 2) appeared in Europe for the first time in 2006 in Russia and Italy and it became predominant in North and Central Europe the year after. In 2007, this sublineage was detected in the Czech Republic, France, Germany, Romania, Poland, Great Britain and Russia. During the same year, clade 2.2.1 and 2.2 sublineage II apparently disappeared from the continent. Clade 2.2 sublineage I also declined, only being notified in 2 epidemiologically linked outbreaks caused by identical viruses in Hungary and the UK [40].

In 2008, five countries reported outbreaks of HPAI A/H5N1 in poultry/wild birds in Europe (Germany, Russia, Switzerland, Ukraine, UK) [4–12]. Based on the available sequences, previously circulating clades and sublineages disappeared from Europe, with the only exception of clade 2.2 sublineage III that was responsible for the outbreaks in the UK (Figure 1) and the Ukraine (based on the analysis of the two partial sequences of 184 and 248 nucleotides available in GenBank; data not shown).

It is interesting to note that a similar situation was described for Nigeria. In this African country, viruses circulating in 2006 and 2007 (clades 2.2.1, 2.2 I and 2.2 II) apparently disappeared, while in 2008 clade 2.2 sublineage III was detected for the first time [41].

Sequences available for 2008 in Russia indicate that viruses of clade 2.3.2, commonly circulating in Eastern Asia, were involved in the outbreak (Figure 1). Sequences for the remaining two countries were not available at the time of writing.

Focusing on the European continent, at present only Germany and Russia reported cases of HPAI A/H5N1 in 2009 in wild birds. Sequences available from viruses isolated in Russia (Tyva Republic) indicate the persistence in the wild bird population of the Asian clade 2.3.2 (Figure 1, Table 2).

Therefore, in the Russian Federation there was no reporting concerning the circulation of clades 2.2 and 2.2.1 from 2008 to July 2009.

### 5.2. Spatial migration of H5N1 virus in Europe

As indicated by the phylogenetic analysis, A/H5N1 viruses collected from 19 European countries are thoroughly interspersed in the ML tree inferred for the HA gene (Figure 1), indicating widespread viral gene flow throughout Europe.

Migration among geographic regions was inferred for the HA gene using a parsimony based method [42] on the ML tree using the program PAUP* [39] using the approach described in previous publications [43]. In this analysis isolates were classified according to the state from which they originated.

Russia, Romania, Germany/Denmark and Hungary/Slovakia served as the largest sources for the spread of the H5 gene in Europe. In particular migration events were observed from Russia to the Ukraine and Italy; from Romania to Russia, Hungary, Slovakia, Croatia, Slovenia, Austria; from Germany/Denmark to Sweden, France, Switzerland, the Czech Republic and the UK; from Hungary/Slovakia to the Czech Republic and Germany (data not shown). This result suggests that migration occurs predominantly from one given country or area to the adjacent geographic regions and mainly from east to west. In some instances, e.g. Russia and Romania, gene movements in the opposite direction can also be observed as well as “in and out” gene migrations in Northern and Central European countries, such as Denmark, Germany and Hungary and the Czech Republic, probably through migratory birds as also indicated in previous studies [24,25,27,31,37,44].

On a continental scale, gene flow was revealed from Asia (we considered primarily the Middle East and Central Asia) to Russia, from Romania to Africa and in the opposite direction, therefore confirming the occurrence of relevant gene movement in opposite directions among the three continents.

## 6. Conclusions

The results illustrated in the last section of this paper derive from the analysis of virtually all the publicly available sequences of HPAI A/H5N1 from Europe and therefore can be considered comprehensive.

However, they are not complete and therefore not definitive due to the unavailability of virus sequences from some infected countries or to the under representation of sequences deposited from certain countries (for example, Turkey or the Ukraine) which could have played a crucial role in the epidemiology and evolution of HPAI A/H5N1 in Europe.

Nevertheless, some key issues and relevant results from this paper can be highlighted and discussed. For the first time since 2007 [11], the phylogeny and the evolution of HPAI A/H5N1 in Europe has been reviewed and revisited with a comprehensive approach in an attempt to harmonize the results derived by the several, valuable scientific papers published previously and listed in this paper. Furthermore, spatial gene migration analysis was for the first time applied on European sequences to better understand virus circulation on this continent and to determine the origin of the viruses entering Europe.

Unequivocally, as already described singularly for specific countries, we showed that genetically distinguishable HPAI A/H5N1 viruses belonging to the main clade 2.2 were introduced into Europe in 2005–2006 and co-circulated. In this period the viruses spread from East to West, following the main flow originating in North-Western China and Central Asia. Although genetic diversity of clade 2.2 can be traced back to the first outbreak at Qinghai lake in April 2005 [9], our findings highlight the significant role of the Russian territory and perhaps other Eastern European countries as the source of the majority of the early viruses spreading into the rest of Europe.

The spatial migration analysis presented herein indicates gene movements from Russia and Romania into other European countries but also in the opposite direction and within Central/Northern Europe. Coupling the data emerging from the phylogenetic and migrational analysis to the time of events, the trade restrictions imposed at the EU borders and within EU member states and to the type and number of outbreaks that occurred in many European countries, the role of wild birds in the introduction and spread of this virus in Europe becomes relevant, with some documented exceptions [21–22]. Also, indications of discrete gene movements from Russia to Asia and particularly from Africa to Europe support the hypothesis of virus gene movements linked to wild birds migrations. This is in agreement with previous findings concerning Europe [44] and, more recently, Africa [15].

Interestingly, based on the sequences available and included in this study it seems that clade 2.2 is disappearing from Europe, with only two documented outbreaks occurring in 2008 caused by 2.2 sublineage III, similarly to what has been described for Nigeria in the same year [41].

However, the recent outbreaks of HPAI A/H5N1 in wild birds registered in Mongolia and in the Tyva Republic (close to Mongolia and Kazakhstan) in May–August 2009 demonstrated the perpetuation of the viruses in these area and in the wild bird population, mimicking the situation described in 2005. Whether clade 2.3.2 viruses responsible for these outbreaks in Central Asia, at least based on the sequences so far deposited, will spread to Europe through migrating wild birds is difficult to predict and probably will depend on several ecological factors and environmental conditions. Certainly, continuous monitoring of the field situation and of the virus evolution will be essential to reveal the introduction of the infection and to control it at the very initial stage, preventing economic losses and public health hazards. However, this is possible only through the continuous support of the veterinary and public health services and transparent sharing of information and data.

## Figures and Tables

**Table 1.** Countries reporting HPAI A/H5N1 outbreaks in poultry and wild birds on the European continent: January 2004 to June 2009.

**Country**	**Animal category**	No. outbreaks in poultry**
Albania	Poultry	3
Austria	Wild birds	
Belgium	Wild birds (illegal trade)	
Bosnia Herzegovina	Wild birds	
Bulgaria	Wild birds	
Croatia	Wild birds	
Czech Republic	Poultry; Wild birds	4
Denmark	Poultry; Wild birds	1
France	Poultry ; Wild birds	1
Germany	Poultry; Wild birds	8
Greece	Wild birds	
Hungary	Poultry; Wild birds	9
Italy	Wild birds	
Poland	Poultry; Wild birds	10
Romania	Poultry; Wild birds	163
Russia	Poultry; Wild birds	149
Serbia & Montenegro	Poultry; Wild birds	1
Slovakia	Wild birds	
Slovenia	Wild birds	
Spain	Wild birds	
Sweden	Poultry; Wild birds	1
Switzerland	Wild birds	
Ukraine	Poultry; Wild birds	42
United Kingdom	Poultry; Wild birds	3

** According to OIE report (www.oie.int; accessed 22/7/2009).

**Table 2.** Genetic diversity of HPAI A/H5N1 in Europe. Only countries whose sequences are publicly available are included in this list.

**Country**	**Year**					
	**2004**	**2005**	**2006**	**2007**	**2008**	**2009**
Austria			**2.2.1**			
Belgium	**1**					
Croatia		**2.2** Sub II				
Czech Republic			**2.2.1**	**2.2** Sub III		
Danimark			**2.2** Sub II			
France			**2.2** Sub I	**2.2** Sub III		
Germany			**2.2** Sub I **2.2** Sub II **2.2.1**	**2.2** Sub III		
Hungary			**2.2** Sub I **2.2** Sub II **2.2.1**	**2.2** Sub I		
Italy			**2.2.1** **2.2** Sub III			
Romania		**2.2.1** **2.2** Sub I **2.2** Sub II	**2.2.1** **2.2** Sub I **2.2** Sub II	**2.2** Sub III		
Russia		**2.2** Sub I **2.2** Sub II **2.2.1**	**2.2** Sub III	**2.2** Sub III	**2.3.2**	**2.3.2**
Slovakia			**2.2.1**			
Slovenia			**2.2.1** **2.2** Sub I			
Sweden			**2.2** Sub II **2.2.1**			
Switzerland			**2.2** Sub I			
Ukraine		**2.2** (undefined sublineage)			**2.2** Sub III	
UK			**2.2** Sub II	**2.2** Sub I **2.2** Sub III	**2.2** Sub III	
Polonia			**2.2** Sub II	**2.2** Sub III		
Spain			**2.2.1**			

## References

[1] Xu X, Subbarao K, Cox NJ, Guo Y (1999). Genetic characterization of the pathogenic influenza A/Goose/Guangdong/1/96 (H5N1) virus: similarity of its hemagglutinin gene to those of H5N1 viruses from the 1997 outbreaks in Hong Kong. Virology.

[2] Capua I, Alexander DJ (2002). Avian influenza and human health. Acta Trop.

[3] Sims LD, Brown IH, Swayne DE (2008). Avian Influenza.

[4] World Health Organization H5N1 avian influenza: timeline of major events. http://www.who.int/csr/disease/avian_influenza/ai_timeline/en/index.html.

[5] Guan Y, Smith GJ, Webby R, Webster RG (2009). Molecular epidemiology of H5N1 avian influenza. Rev Sci Tech.

[6] Li KS, Guan Y, Wang J, Smith GJ, Xu KM, Duan L, Rahardjo AP, Puthavathana P, Buranathai C, Nguyen TD, Estoepangestie AT, Chaisingh A, Auewarakul P, Long HT, Hanh NT, Webby RJ, Poon LL, Chen H, Shortridge KF, Yuen KY, Webster RG, Peiris JS (2004). Genesis of a highly pathogenic and potentially pandemic H5N1 influenza virus in eastern Asia. Nature.

[7] Vijaykrishna D, Bahl J, Riley S, Duan L, Zhang JX, Chen H, Peiris JS, Smith GJ, Guan Y (2008). Evolutionary dynamics and emergence of panzootic H5N1 influenza viruses. PLoS Pathog.

[8] World Health Organization/World Organisation for Animal Health/Food and Agriculture Organization H5N1 Evolution Working Group Toward a unified nomenclature system for highly pathogenic avian influenza virus (H5N1) [conference summary]. Emerg. Infect. Dis..

[9] Chen H, Li Y, Li Z, Shi J, Shinya K, Deng G, Qi Q, Tian G, Fan S, Zhao H, Sun Y, Kawaoka Y (2006). Properties and dissemination of H5N1 viruses isolated during an influenza outbreak in migratory waterfowl in western China. J Virol.

[10] Editorial team (2005). Highly pathogenic avian influenza reported in Russian bird populations. Euro Surveill.

[11] Salzberg SL, Kingsford C, Cattoli G, Spiro DJ, Janies DA, Aly MM, Brown IH, Couacy-Hymann E, De Mia GM, Dung Do H, Guercio A, Joannis T, Maken Ali AS, Osmani A, Padalino I, Saad MD, Savić V, Sengamalay NA, Yingst S, Zaborsky J, Zorman-Rojs O, Ghedin E, Capua I (2007). Genome analysis linking recent European and African influenza (H5N1) viruses. Emerg Infect Dis.

[12] World Organisation for Animal Health (OIE) Avian Influenza. http://www.oie.int/eng/info_ev/en_AI_avianinfluenza.htm.

[13] World Health Organization http://www.who.int/csr/disease/avian_influenza/country/cases_table_2009_08_11/en/index.html.

[14] WHO/OIE/FAO H5N1 Evolution Working Group (2009). Continuing progress towards a unified nomenclature for the highly pathogenic H5N1 avian influenza viruses: divergence of clade 2.2 viruses. Influenza Other Respi Viruses.

[15] Cattoli G, Monne I, Fusaro A, Joannis TM, Lombin LH, Aly MM, Arafa AS, Sturm-Ramirez KM, Couacy-Hymann E, Awuni JA, Batawui KB, Awoume KA, Aplogan GL, Sow A, Ngangnou AC, El Nasri Hamza IM, Gamatié D, Dauphin G, Domenech JM, Capua I (2009). Highly pathogenic avian influenza virus subtype H5N1 in Africa: a comprehensive phylogenetic analysis and molecular characterization of isolates. PLoS One.

[16] Lipatov AS, Evseenko VA, Yen HL, Zaykovskaya AV, Durimanov AG, Zolotykh SI, Netesov SV, Drozdov IG, Onishchenko GG, Webster RG, Shestopalov AM (2007). Influenza (H5N1) viruses in poultry, Russian Federation, 2005–2006. Emerg Infect Dis.

[17] Lvov DK, Kaverin NV, Klenk H-D, Matrosovich MN, Stech J (2008). Avian Influenza.

[18] Coulombier D, Paget WJ, Meijer A, Ganter B (2005). Highly pathogenic avian influenza reported to be spreading into western Russia. Euro Surveill.

[19] Anonym (2005). Avian influenza suspected in Turkey: EC restricts imports. Vet Rec.

[20] Anonym (2005). H5N1 avian influenza virus reaches Europe. Vet Rec.

[21] Van Borm S, Thomas I, Hanquet G, Lambrecht B, Boschmans M, Dupont G, Decaestecker M, Snacken R, van den Berg T (2005). Highly pathogenic H5N1 influenza virus in smuggled Thai eagles, Belgium. Emerg Infect Dis.

[22] Brown I Overview of animal outbreaks of AI H5N1 globally since 1997. http://www.fao.org/docs/eims/upload//250653/aj140e00.pdf.

[23] Bogner P, Capua I, Cox NJ, Lipman DJ (2006). A global initiative on sharing avian flu data. Nature.

[24] Bragstad K, Jørgensen PH, Handberg K, Hammer AS, Kabell S, Fomsgaard A (2007). First introduction of highly pathogenic H5N1 avian influenza A viruses in wild and domestic birds in Denmark, Northern Europe. Virol J.

[25] Weber S, Harder T, Starick E, Beer M, Werner O, Hoffmann B, Mettenleiter TC, Mundt E (2007). Molecular analysis of highly pathogenic avian influenza virus of subtype H5N1 isolated from wild birds and mammals in northern Germany. J Gen Virol.

[26] Rinder M, Lang V, Fuchs C, Hafner-Marx A, Bogner KH, Neubauer A, Büttner M, Rinder H (2007). Genetic evidence for multi-event imports of avian influenza virus A(H5N1) into Bavaria, Germany. J Vet Diagn Invest.

[27] Starick E, Beer M, Hoffmann B, Staubach C, Werner O, Globig A, Strebelow G, Grund C, Durban M, Conraths FJ, Mettenleiter T, Harder T (2008). Phylogenetic analyses of highly pathogenic avian influenza virus isolates from Germany in 2006 and 2007 suggest at least three separate introductions of H5N1 virus. Vet Microbiol.

[28] Harder TC, Teuffert J, Starick E, Gethmann J, Grund C, Fereidouni S, Durban M, Bogner KH, Neubauer-Juric A, Repper R, Hlinak A, Engelhardt A, Nöckler A, Smietanka K, Minta Z, Kramer M, Globig A, Mettenleiter TC, Conraths FJ, Beer M (2009). Highly pathogenic avian influenza virus (H5N1) in frozen duck carcasses, Germany, 2007. Emerg Infect Dis.

[29] Globig A, Staubach C, Beer M, Köppen U, Fiedler W, Nieburg M, Wilking H, Starick E, Teifke JP, Werner O, Unger F, Grund C, Wolf C, Roost H, Feldhusen F, Conraths FJ, Mettenleiter TC, Harder TC (2009). Epidemiological and ornithological aspects of outbreaks of highly pathogenic avian influenza virus H5N1 of Asian lineage in wild birds in Germany, 2006 and 2007. Transbound Emerg Dis.

[30] Gall-Reculé GL, Briand FX, Schmitz A, Guionie O, Massin P, Jestin V (2008). Double introduction of highly pathogenic H5N1 avian influenza virus into France in early 2006. Avian Pathol.

[31] Nagy A, Machova J, Hornickova J, Tomci M, Nagl I, Horyna B, Holko I (2007). Highly pathogenic avian influenza virus subtype H5N1 in Mute swans in the Czech Republic. Vet Microbiol.

[32] Nagy A, Vostinakova V, Pindova Z, Hornickova J, Cernikova L, Sedlak K, Mojzis M, Dirbakova Z, Machova J (2009). Molecular and phylogenetic analysis of the H5N1 avian influenza virus caused the first highly pathogenic avian influenza outbreak in poultry in the Czech Republic in 2007. Vet Microbiol.

[33] Irvine RM, Banks J, Londt BZ, Lister SA, Manvell RJ, Outtrim L, Russell C, Cox WJ, Ceeraz V, Shell W, Landeg FJ, Wilesmith JW, Alexander DJ, Brown IH (2007). Outbreak of highly pathogenic avian influenza caused by Asian lineage H5N1 virus in turkeys in Great Britain in January 2007. Vet Rec.

[34] Kiss I, Gyarmati P, Zohari S, Ramsay KW, Metreveli G, Weiss E, Brytting M, Stivers M, Lindström S, Lundkvist A, Nemirov K, Thorén P, Berg M, Czifra G, Belák S (2008). Molecular characterization of highly pathogenic H5N1 avian influenza viruses isolated in Sweden in 2006. Virol J.

[35] Barral M, Alvarez V, Juste RA, Agirre I, Inchausti I (2008). First case of highly pathogenic H5N1 avian influenza virus in Spain. BMC. Vet Res.

[36] Hofmann MA, Renzullo S, Baumer A (2008). Phylogenetic characterization of H5N1 highly pathogenic avian influenza viruses isolated in Switzerland in 2006. Virus Genes.

[37] Szeleczky Z, Dán A, Ursu K, Ivanics E, Kiss I, Erdélyi K, Belák S, Muller CP, Brown IH, Bálint A (2009). Four different sublineages of highly pathogenic avian influenza H5N1 introduced in Hungary in 2006–2007. Vet Microbiol.

[38] Ducatez MF, Olinger CM, Owoade AA, De Landtsheer S, Ammerlaan W, Niesters HG, Osterhaus AD, Fouchier RA, Muller CP (2006). Avian flu: multiple introductions of H5N1 in Nigeria. Nature.

[39] Wilgenbusch JC, Swofford D (2003). Inferring evolutionary trees with PAUP*. Curr Protoc Bioinformatics.

[40] Anonym (2007). Viruses responsible for avian influenza in Suffolk and Hungary “essentially identical”. Vet Rec.

[41] Fusaro AM, Joannis T, Monne I, Salviato A, Yakubu B, Meseko C, Oladokun T, Fassina S, Capua I, Cattoli G (2009). Introduction into Nigeria of a distinct genotype of avian influenza virus (H5N1). Emerg Infect Dis.

[42] Nakano T, Lu L, Liu P, Pybus OG (2004). Viral gene sequences reveal the variable history of hepatitis C virus infection among countries. J Infect Dis.

[43] Carrington CV, Foster JE, Pybus OG, Bennett SN, Holmes EC (2005). Invasion and maintenance of dengue virus type 2 and type 4 in the Americas. J Virol.

[44] Kilpatrick AM, Chmura AA, Gibbons DW, Fleischer RC, Marra PP, Daszak P (2006). Predicting the global spread of H5N1 avian influenza. Proc Natl Acad Sci U S A.

